# The Impact of Negative Mood on Event-Related Potentials When Viewing Pornographic Pictures

**DOI:** 10.3389/fpsyg.2021.673023

**Published:** 2021-07-05

**Authors:** Charlotte Markert, Andreas M. Baranowski, Simon Koch, Rudolf Stark, Jana Strahler

**Affiliations:** ^1^Department of Psychotherapy and Systems Neuroscience, Psychology and Sport Science, University of Giessen, Giessen, Germany; ^2^Bender Institute of Neuroimaging, University of Giessen, Giessen, Germany; ^3^Center for Mind, Brain and Behavior, Universities of Marburg and Giessen, Giessen, Germany

**Keywords:** event-related potentials, motivated attention, negative affect, pornography addiction, pornography use, sexual cue reactivity

## Abstract

**Background:** Negative affective states may increase the risk for problematic pornography use. Underlying neurophysiological mechanisms are, however, not completely understood. Previous research suggests that the participants' emotional state may affect neural processing of sexual stimuli. The aim of this study was to investigate neural correlates of negative affect-induced alterations in sexual cue reactivity in healthy men. The moderating effects of habitual porn consumption, trait sexual motivation, and symptoms of cybersex addiction were also considered.

**Method:** Sixty-four healthy men engaged in a sexual cue reactivity task (passive viewing of explicit sexual pictures and neutral pictures depicting scenes of social interaction) during negative (*n* = 32) vs. neutral affect (*n* = 32), induced via tailored feedback on a performance task. Self-reported sexual arousal and event-related brain potentials indicated cue reactivity and motivated attention. Symptoms of cybersex addiction and trait sexual motivation were assessed with the help of the short Internet Addiction Test, adapted to online sexual activities, and the Trait Sexual Motivation Questionnaire.

**Results:** Negative feedback increased negative affect after the performance task. While sexual pictures compared to neutral pictures elicited significantly larger P300 and late positive potential (LPP) amplitudes, there was no general effect of negative feedback on sexual stimuli-related P300 and LPP amplitudes. In the negative feedback group, men with higher solitary sexual motivation levels showed higher P300/LPP difference amplitudes for sexual stimuli compared to men with lower levels of solitary sexual motivation. The opposite effect was found in the group with neutral feedback. There was no link to other aspects of trait sexual motivation and symptoms of cybersex addiction.

**Conclusions:** Results suggest that higher levels of solitary sexual motivation may enhance motivated attention toward sexual stimuli among men receiving negative performance feedback. Other characteristics of sexual behaviors and traits provided no exploratory value. Future studies extending onto men suffering from compulsive sexual behavior disorder will have to closer look at the neurophysiological bases of why and when some men develop an addictive pornography consumption.

## Introduction

Digitalization has an impact on a wide range of areas of life, including sexual behaviors. Through mobile devices such as tablets or smartphones it is possible to consume pornography almost anytime and everywhere. Cooper (1998) described the three key characteristics of the Internet (“Triple-A Engine”: access, affordability, and anonymity), which increase the attraction and usage of internet pornography. Today, men between the ages of 35–50 are the largest consumer group (Blais-Lecours et al., 2016; Rissel et al., 2017), but regular usage is also seen among other groups including women (Baranowski et al., 2019; Herbenick et al., 2020), younger adults and adolescents from various socio-economic backgrounds (Mattebo et al., 2013, 2016; Bothe et al., 2020b). This previous research is not free from methodological shortcomings such as small and biased samples or questionable validity of assessment measures. Still, the increase of pornography consumption, particularly among younger people (Price et al., 2016), goes along with rising numbers of individuals who report a problematic or an addictive use with psychopathological consequences (Duffy et al., 2016). This calls for increased efforts to better understand the transition from a recreational to a pathological use, and to provide improved preventive and therapeutic interventions.

Several reviews have shown that, among healthy and clinical samples, visual sexual stimuli are perceived as highly rewarding (Gola et al., 2016b) and that brain areas associated with reward learning respond to sexual stimuli in a similar manner as they respond to other biologically relevant stimuli (Georgiadis and Kringelbach, 2012; Stoléru et al., 2012; Poeppl et al., 2014; Strahler et al., 2018). While recreational pornography use focusses on experiencing satisfaction (Ross et al., 2012; Gola et al., 2016a; Wordecha et al., 2018), some people develop a shift toward over-frequent, uncontrolled use, which persists even though it causes disadvantages in interpersonal relationships or for work or leisure activities. If this reaches a clinically relevant level, Compulsive Sexual Behavior Disorder (CSBD) can be diagnosed according to the new classification in ICD-11 (World Health Organization, 2018). Main characteristic of CSBD is the persistent inability to control intense, repetitive sexual impulses or urges resulting in recurring sexual behavior. This pattern negatively affects personal, familial, social, educational and/or occupational areas of functioning. Indeed, the vast majority of consumers show a purely recreational use and problematic symptoms are exhibited in only a small minority. Most studies show a male preponderance regarding prevalence rates for CSBD with a sex ratio of ~1:4 (prevalence in women: 1–3%; prevalence in men: 4–11%; Rissel et al., 2017; Grubbs et al., 2019; Bothe et al., 2020a; Kowalewska et al., 2020). Studies show that addictive internet pornography use (for which there are numerous other terms, e.g., excessive pornography use, pornography addiction, pathological pornography use, etc.) is reported as the most frequent manifestation of CSBD (Reid et al., 2012; Engel et al., 2019a; Bothe et al., 2020a). In the present work, the term “addictive internet pornography use” is therefore used, except when similar but different constructs are addressed.

Different models have been proposed to better understand mechanisms underlying addictive pornography use, including models of motivation, emotion regulation, stage models, and integrated models. Reid et al. (2011) outline typical motives for pornography use, that is emotional avoidance, sexual curiosity, excitement seeking and sexual pleasure. In their sample of treatment-seeking hypersexual men, emotional avoidance, that is to use pornography to avoid unpleasant feelings and relieve stress, correlated positively with trait measures for emotional distress, anxiety, depression, and impulsiveness. This association has since been confirmed by other survey studies in non-treatment seeking young adults and men with hypersexual disorder (Engel et al., 2019b; Pettorruso et al., 2020). In a longitudinal study with adolescents, higher baseline levels for negative emotions and impulsivity predicted addictive pornography use 3 years later (Rousseau et al., 2020). Another study investigated changes of mood and sexual arousal before and after self-determined internet pornography use in a non-clinical male sample (Laier and Brand, 2017). The results showed usage-related mood and arousal changes suggesting reinforcing effects of pornography use. Additionally, participants with a stronger tendency toward cybersex addiction reported a generally worse mood, but greater mood increases were positively linked to the degree of addictive pornography use (Laier and Brand, 2017). Overall, data support theories which suggest emotional instability, impaired stress regulation and impulse control as a prominent pattern in hypersexual individuals (Reid et al., 2014).

The Interaction of Person-Affect-Cognition-Execution (I-PACE) model of specific internet-use disorders (Brand et al., 2016, 2019) distinguishes between earlier and later phases of addiction development with emotion regulation playing a pivotal role in all of them. In earlier phases, pornography use contributes to gratification (e.g., in the form of pleasure). Based on this experience, pornography use may be increasingly used for emotion regulation and thus becomes a reinforcing or perpetuating mechanism of pornography use (Laier and Brand, 2017). Through repeated and possibly increased pornography use, conditioning processes strengthen associations between external triggers and affective/cognitive responses which may lead to compensatory pornography use in response to negative affect and craving in later stages of addiction development.

Different models of addiction development [see also A-B-C model of hypersexual disorder from Stein (2008), or the Brain Disease Model of Addiction from Volkow et al. (2016)] emphasize and specify the role of neural substrates for emotion regulation and adaptation to negative affect and stress in the development of addiction. Research on neurobiological and neural underpinnings of (addictive) pornography use is, however, still rare. Initial neurobiological studies in individuals with addictive sexual behaviors suggest hypothalamic pituitary adrenal (HPA) axis dysregulation (Chatzittofis et al., 2016), oxytocin signaling (Boström et al., 2020), and heightened neuroinflammation (Jokinen et al., 2017) as possible pathophysiological mechanisms. In terms of neural correlates, studies focused on the activity in brain regions located in the reward network and the limbic system. Such studies revealed similar activation patterns for sexual stimuli as for other drug stimuli (Love et al., 2015; Gola et al., 2016b; Kühn and Gallinat, 2016). Longer hours of internet pornography use correlated with lower gray matter volume in the right caudate nucleus and lower activity to sexual images in the left putamen in healthy men (Kühn and Gallinat, 2014). According to the authors, these findings may indicate a tolerance that has developed due to desensitization. Even fewer studies compared individuals with and without addictive pornography use. Voon et al. (2014) showed higher anterior cingulate cortex (ACC), ventral striatal, and amygdala activity as well as higher functional connectivity of the ACC–striatal–amygdala network during watching of sexually explicit cues in hypersexual individuals. Subjective sexual arousal ratings of pornographic pictures (Brand et al., 2011) as well as the ventral striatal response to preferred pornographic pictures were predicted by symptoms of cybersex addiction as measured by the short Internet Addiction Test Adapted to Online Sexual Activities (s-IATsex). A study by Klucken et al. (2016) found increased amygdala activity during appetitive conditioning with sexual stimuli in men with compulsive sexual behavior vs. healthy controls. This may be indicative of emotional dysregulation in individuals with addictive pornography use. In comparison, the results of a study by Gola et al. (2017) indicate that the anticipation of pornographic pictures is associated with stronger activation of the ventral striatum in persons with addictive pornography use, but not the response to the pornographic pictures themselves.

In addition to fMRI results, electroencephalography (EEG) studies confirm the arousing and motivationally salience nature of pornographic pictures. Presentation of those pictures compared to neutral pictures lead to heightened positive EEG components known to reflect stimulus salience and attentive processing, namely the P300 and the Late Positive Potential (LPP) in both healthy individuals and individuals self-identifying as having problems with regulating their sexual stimuli consumption (van Lankveld and Smulders, 2008; Steele et al., 2013; Prause et al., 2015a). While the P300 appears 300–500 ms post stimulus and is partly generated in the ACC, thus best measurable at centroparietal recording sites, the LPP extends beyond this and reflects sustained increases in attention (Hajcak et al., 2010). The LPP can best be localized at central, parietal, and occipital sites (Foti et al., 2009). Enhanced P300 and LPP elicited by addictive cues are well-replicated in other substance-related and behavioral addictions (Dunning et al., 2011; Wölfling et al., 2011). Such a hypersensitivity to pornographic pictures has been proposed to underlie addictive pornography use (Voon et al., 2014; Brand et al., 2019). The only two studies using EEG to investigate individuals with self-reported addictive pornography use, could however not find support for this assumption. In one study, addictive pornography use was unrelated to the P300 (Steele et al., 2013). Another study of the same research group found lower LPP amplitudes after passively watching pornographic pictures in individuals with addictive pornography use compared to healthy controls (Prause et al., 2015b).

In the search for possible psychological correlates of sexual cue reactivity and risk factors of addictive pornography use, symptoms of cybersex addiction, habitual porn use and trait sexual motivation appear most relevant given available literature. As described above, prolonged pornography use in healthy men is associated with lower activity in response to pornographic pictures in the left putamen (Kühn and Gallinat, 2014). Whether something similar is also seen in CSBD individuals still remains to be investigated. Concerning sexual motivation, the findings are heterogeneous. In an all-female healthy sample, Demos et al. (2012) showed a positive association between higher sexual motivation and greater sexual cue reactivity in the brain's reward networks. By contrast, the P300 was inversely related to sexual motivation in individuals with addictive pornography use (Steele et al., 2013). However, the authors did not examine sexual motivation as a trait but as a current state. In a previous study in healthy men and women, our group demonstrated that trait sexual motivation correlated with nucleus caudatus activity (but no other ROI) when watching pornographic pictures (Strahler et al., 2018). But there were no significant associations between trait sexual motivation, the extent of porn use and neural responses of the nucleus accumbens toward pornographic pictures in healthy men (Stark et al., 2019).

To summarize, studies implicate considerably similar brain regions involved in addictive pornography use that is reward-associated brain regions like the striatum and nucleus accumbens, but also the amygdala, hippocampus, and hypothalamus. Whether there is also a critical role for the temporal dynamics underlying salience formation and attention allocation to sexual stimuli remains unclear. Evidence of emotion regulatory use of pornography playing a role in addiction development implies that a negative affective state may cater into the salience of and attention directed at pornographic material. Yet, negative mood related alterations in sexual cue reactivity as indicated by electrocortical potentials have not been studied. The purpose of the current study was therefore to investigate the neural correlates of negative affect-induced alterations in sexual cue reactivity in healthy heterosexual men via recording electro-cortical brain response during watching pornographic pictures. We hypothesized that negative affect will induce higher motivational attention, operationalized through the EEG parameters LPP and P300. Our second aim was to examine the moderating effects of habitual porn use, trait sexual motivation, and symptoms of cybersex addiction. Our hypothesis was that these moderators influence cue reactivity to pornographic pictures under negative affect. This may provide further evidence for the relevance of affect regulation in the development and maintenance of addictive porn use and CSBD.

## Materials and Methods

### Participants

Healthy male adults were recruited for this EEG study via university e-mail newsletters, social media advertisement, and by directly addressing pedestrians on the university campus. The study was publicly labeled as study on “General cognitive ability and processing of sexual images.” Affect manipulation was not mentioned to the applicants. The true aims of the study were explained after completion of all experimental procedures. Eligibility criteria were proficiency in the German language to ensure comprehension of instructions and questionnaires, male gender, absence of acute or anamnestic psychological, neurological, or somatic disorders and medication, right-handedness, body-mass-index between 18 and 35 kg/m^2^, age between 18 and 45 years to minimize the impact of age-related changes in EEG (Hashemi et al., 2016), sexual attraction to females, and no daily alcohol consumption or illegal substance use within the last 6 months. Further, volunteers who underwent diagnostic assessment of their general cognitive ability in the past were excluded to ensure effectiveness of the affect manipulation using tailored feedback on a test for general cognitive ability. Usual EEG contraindications applied (being unable to sit still for a long time, wearing a pacemaker, hearing aids, suffering from claustrophobia, bald head or rasta curls). Initially, 72 men (mean age 24.81 years) were recruited into this trial and completed all study procedures. Due to poor EEG data quality, *n* = 8 data sets (*n* = 4 negative affect condition, *n* = 4 control condition) had to be excluded resulting in a final sample of 64 (mean age 24.94 years). Excluded participants did not differ significantly in age, body-mass-index, relationship status or any of the analyzed moderators from the remaining sample (all *p* > .05, using Mann-Whitney-U test and χ^2^-test, respectively). Participation was voluntary and all participants provided written informed consent. Data was collected in pseudonymized form with individual code words. Participants were compensated with either 8.00€ per hour or course credits. All experimental procedures were conducted in compliance with national legislation and the Declaration of Helsinki and approved by the local ethics committee (reference number: 2019-0005).

### Procedures and Design

Interested men were first contacted *via* phone for the screening of eligibility criteria. Eligible men were invited for individual laboratory assessment and asked to avoid use of hair spray/hair gel on the date of the assessment. Study procedures took ~2 h and were conducted by two male experimenters in a quiet windowless room. Participants were randomly assigned into two equal-sized groups with one group undergoing negative affect induction and the other one remaining in neutral mood. Affect manipulation was conducted with tailored feedback on a test for general cognitive ability. Except for the differently tailored feedback, procedures were the same for both groups. Participants were neither aware which group they were assorted to nor that the cognitive test targeted at affect manipulation.

**Figure 1:**
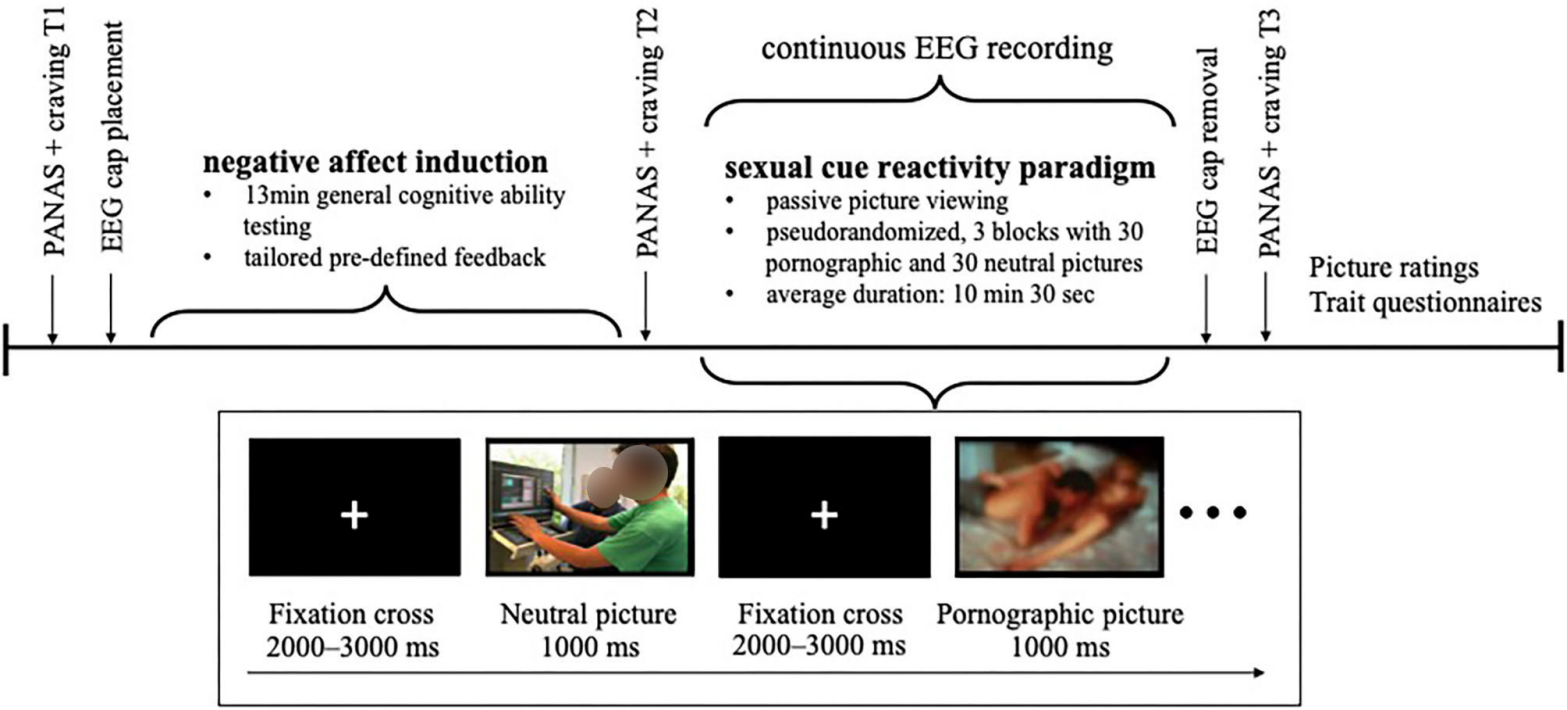
Depiction of the experimental protocol.

After giving informed consent, momentary affect as well as craving for pornography and masturbation was assessed as a baseline. Participants were prepared for EEG recording. Hereafter, affect manipulation was conducted by means of a negative feedback paradigm as described below. Subsequently, momentary affect and craving were assessed a second time. Participants, then, viewed the sexual cue reactivity paradigm while EEG was continuously recorded. Followingly, the EEG cap was removed and after that, momentary affect and craving were assessed a third time, stimuli were rated, and several questionnaires were answered by the participants. Finally, participants were compensated for participation, they were clarified about the tailored feedback and that their general cognitive ability has not been evaluated for real. In this context, they were asked whether they questioned their test result during the examination and believed the cover story for the study (yes/no). A schematic depiction of the experimental protocol is shown in Figure 1.

### Affect Manipulation

Participants completed three subtests of a German diagnostic tool for assessing general cognitive ability (Leistungsprüfsystem 2, LPS-2; Kreuzpointner et al., 2013). To mime an authentic execution, the LPS-2 subtests were conducted as instructed by the test manual. Completion of the subtests took 13 min in total. Participants' performance, however, was not evaluated in real terms. After a latency of 2 min, the participants were handed a predefined standardized feedback sheet. In the negative affect condition, participants received negative feedback, that is they received a feedback sheet indicating they had scored 2.9 out of 10.0 points coupled with the verbal statement that the result was below average. Participants in the neutral feedback group (neutral condition) received a feedback sheet indicating 6.8 out of 10.0 points coupled with the verbal statement that their result was slightly above average.

### Sexual Cue Reactivity Paradigm

Thirty neutral and thirty pornographic pictures were presented in a passive viewing paradigm programmed with Presentation Version 20.0 (Neurobehavioral System Inc., Albany, CA, USA). All pictures were 31 cm wide, presented in color on a black background on a 27-inch monitor with 1,920 × 1,080 pixel resolution. All stimuli were derived from Kagerer et al. (2014). Pornographic pictures showed one male and one female adult engaging in partnered sexual behavior, that is oral, vaginal, or anal intercourse. No fetish-relevant content was depicted. In half of the pornographic pictures genitalia were clearly visible whereas in the other half genitalia were masked by bodies or objects. Female breasts were visible in all sexual pictures. Neutral pictures showed two or more fully clothed adult(s) in an everyday situation (e.g., engaging in conversation). Pictures were presented in three blocks. Each block contained all pictures in randomized order with the restriction that the same stimulus category could be presented a maximum of three times in succession. Therefore, each picture was presented three times for 1,000 ms, resulting in 180 stimulus presentations in total. Pictures were intercepted with a jittered presentation of a white fixation cross on black background between 2,000 and 3,000 ms. In total, the paradigm lasted between 9 and 12 min, on average 10 min and 30 s.

### EEG Recording and Event Related Potential Data Reduction

Participants' electrocortical activity was continuously recorded with an active 32-channel amplifier (BrainVision actiChamp, Brain Products GmbH, Gilching, Germany) and 32 Ag/AgCl electrodes (EASYCAP GmbH, Herrsching, Germany). For attachment of electrodes participants wore an EEG cap (EASYCAP GmbH, Herrsching, Germany) which assured location of the electrodes in accordance with the international 10–20 system (Klem et al., 1999). Electrodes were attached to the participants' scalp with an electrolyte paste. Impedances of all electrodes were kept below 10 kΩ. Electrode signal was recorded via BrainVision Recorder software (Brain Products GmbH, Gilching, Germany). Sampling rate was 500 Hz, no software filters were applied during recording. Ground electrode was Fpz and electrodes were referenced at Cz. Line noise was kept at minimum by instructing participants to remain calm and relaxed during the paradigm, and to let their gaze rest on the fixation cross between the image presentations.

Data were processed using BrainVision Analyzer 2.2 software (Brain Products GmbH, Herrsching, Germany). First, a high-pass filter of 0.001 Hz and a low-pass filter of 30 Hz were applied. Data were then corrected for ocular artifacts caused by eye blinks or saccades by computing Independent Component Analysis (ICA) and exclusion of components reflective of ocular artifacts. Following reverse ICA, data were visually inspected for remaining artifacts caused by body movements and corresponding epochs were excluded. EEG channels were then re-referenced to linked mastoid activity (TP9, TP10). Data were segmented, i.e., stimulus-locked from −500 to 2,499 ms post stimulus. Segments were baseline corrected from −500 to 0 ms (stimulus onset) and then averaged for the two image categories separately. On average, 85.43 (*SD* = 6.23) out of 90 segments were used to compute individual ERP. Taking into consideration that P300 and LPP temporal windows do partially overlap (Hajcak et al., 2010), P300 was defined as mean ERP amplitude from 300 to 500 ms and LPP as mean ERP amplitude from 500 to 800 ms post-stimulus onset. Defined intervals are in line with studies examining both ERP components following visual sexual stimuli (van Lankveld and Smulders, 2008; Han et al., 2018). Mean ERP amplitudes in the respective temporal windows were averaged across electrodes CP1, CP2, P3, Pz, P4. Electrode selection was based on visual inspection and is in line with findings, that both ERP components are measurable best at centroparietal recording sites (Hajcak et al., 2010).

### Self-Report Measures

#### Momentary Affect

Momentary affect was assessed with sum scores of the Positive and Negative Affect Schedule (Watson et al., 1988) prior to EEG cap placement, following tailored feedback, and following the sexual cue reactivity paradigm. The two PANAS subscales, namely *positive affect* and *negative affect*, consist of ten items each. The 20 items (negative e.g.,: upset, guilty, distressed, positive e.g.,: excited, inspired, proud) were each rated on a 5-point scale ranging from *1* = “very slightly or not at all” to *5* = “very much.” Internal consistency of the *positive affect* scale was acceptable to satisfying throughout assessment (baseline: Cronbach's α = .771; after affect manipulation: α = 0.814; after the sexual cur reactivity paradigm: α = 0.857) and internal consistency of the *negative affect* scale was acceptable to satisfying throughout assessment as well (baseline: Cronbach's α = .735, after affect manipulation: α = 0.870, after the sexual cur reactivity paradigm: α = 0.835, respectively).

#### Craving

Participants' momentary craving for pornography and masturbation was assessed by one question each (“To what extent do you currently feel the need to consume pornography/masturbate?”) added to the paper-pencil version of the PANAS. The two questions were answered using the same five-point scale ranging from *1* = “very slightly or not at all” to *5* = “very much.”

#### Stimuli Ratings

Following the sexual cue reactivity paradigm, participants rated all pictures on 9-point Likert scales regarding valence (*very unpleasant* to *very pleasant*), arousal (*calm and relaxed* to *very excited*), and sexual arousal (*not at all* to *very much*). Pictures were rated in randomized order across participants. Participants viewed the pictures one by one again for a maximum of 10 s each, which were then succeeded by the three rating scales. Valence and arousal scales were visually anchored with the Self Assessment Manikin Scale (Bradley and Lang, 1994). Rating of sexual arousal was visualized by blocks of increasing size.

#### Trait Measures of Sexual Behaviors

Participants' symptoms of cybersex addiction were assessed with the short version of the Internet Addiction Test (Pawlikowski et al., 2013) adapted to online sexual activities (s-IATsex; Laier et al., 2013). Twelve items (e.g., “How often do you find that you stay on sex sites on the internet longer than you intended?”) were answered on a scale from *1* = “never” to *5* = “very often” resulting in sum scores ranging from 12 to 60. Sum scores exceeding 30 are classified to be indicative of problematic sexual internet use. Internal consistency was acceptable with Cronbach's α = .788. Trait sexual motivation was examined by means of the 45-item Trait Sexual Motivation Questionnaire (TSMQ, Stark et al., 2015). Out of 45 items in total only 35 items are analyzed to compute mean scores of trait sexual motivation. Participants were instructed to indicate to which extent each item described their sexual motivation using a six-point Likert scale ranging from *0* = “not at all” to *5* = “very much.” The analyzed items make up four subscales of trait sexual motivation, namely *Solitary Sexuality* (10 items, Cronbach's α = .856), *Importance of Sex* (15 items, Cronbach's α = .901), *Seeking Sexual Encounters* (4 items, Cronbach's α = .838), and *Comparison with Others* (6 items, Cronbach's α = .871). The scale *Solitary Sexuality* indicates interest in sexual activities independent of a sexual relationship. Most of this scale's items relate to masturbation and the interest to be sexually aroused by pornographic material. The scale *Importance of Sex* includes several items about the need to be sexually active. The scale *Seeking Sexual Encounters* includes items asking about behaviors with the intention to get in contact with new potential sex partners. The scale *Comparison with Others* consists of items asking how an individual perceives their own sexual motivation compared to others'. Participants' answers were averaged across corresponding items to indicate an individual's mean score on each subscale. Participants' mean scores on each subscale were then averaged again to compute an individual's mean trait sexual motivation. Mean trait sexual motivation had excellent internal consistency with Cronbach's α = .918. The single item “How much time did you spend viewing pornographic material within the last month” was used to assess habitual porn use (h/month). Participants were given the option to indicate the time spent viewing pornography either per day, per week, or per month. Answers were then transformed into hours per month (h/month) based on the definitions that a month consists of 30 days, a week of 7 days, a day of 24 h and an hour of 60 min. Sexual orientation of the participants was assessed with the seven-item Kinsey scale (Kinsey et al., 1948) ranging from *0* = “exclusively heterosexual fantasies and behaviors” to *6* = “exclusively homosexual fantasies and behaviors.” All participants but one reported a predominantly heterosexual orientation [Kinsey score *0*: *n* = 52 (81.3%); Kinsey score *1*: *n* = 9 (14.1%); Kinsey score *2*: *n* = 2 (3.1%), Kinsey score *3*: *n* = 1 (1.6%)]. Exclusion of the man indicating heterosexual and homosexual behaviors of about the same frequency (Kinsey score 3; negative affect condition) did not change results. This data set was therefore retained in the final analyses.

An accompanying questionnaire gathered data on age, body mass index (BMI), partnership status, smoking (yes, no), and alcohol consumption measured using the Alcohol Use Disorders Identification Test (AUDIT, Babor et al., 2001; Cronbach's α = .68).

### Statistical Analyses

Descriptive statistics are reported as mean and standard deviation (SD) or numbers and frequency. Group comparisons of continuous data were performed with Student's *t*-test, categorical variables were examined with the Fisher's exact test. To test for differences in sexual cue reactivity, mixed-model ANOVAs were performed with the between-factor group (negative affect, neutral affect) and the within-factor picture category (pornographic, neutral). Separate models were run for each EEG component (P300, LPP) and stimulus rating (valence, arousal, sexual arousal). Effect sizes were reported as Cohen's *d*, φ, and $\eta_{p}^{2}$, respectively.

The moderating effects of habitual porn use, trait sexual motivation, and symptoms of cybersex addiction on neural responses toward pornographic pictures were exploratively evaluated. We used three-stage hierarchical regressions to predict neural responses toward pornographic pictures with group included at step 1, the group mean-centered trait sexual behavior factor (moderator) entered regression at step 2, and step 3 included the group X group mean-centered moderator product term. As criterion, we calculated the difference amplitudes between neural responses toward pornographic pictures minus neutral pictures. Plotting two-way interaction effects using the z-standardized scores and procedures described by Dawson (2014) eased interpretation of the assumed moderation. The relation between P300/LPP and trait sexual behavior factor was plotted for both conditions, neutral and negative feedback. All assumptions for regression analyses were met (linearity assumption; VIF <2.39; Cook's distance <0.02; normally distributed residuals as indicated from normal P-P-plots; homoscedasticity, i.e., no clear distribution pattern in scatterplot of residuals vs. predicted values). Each moderator was tested in a separate model. The level of significance was set at α = 0.05 for all testing. All statistical analyses were carried out using SPSS v.23 for Mac (IBM Statistics, IBM Corporation).

## Results

### Sample Description

The negative feedback group and the neutral feedback group did not differ in age, BMI, partnership, smoking status, alcohol consumption or pornography use (Table 1). The neutral feedback group reported significantly higher scores on the subscale *Importance of Sex* as well as the total score of the TSMQ, and higher arousal during the rating of neutral pictures. Other group comparisons did not reach significance.

**Table 1:** Baseline characteristics of total sample and separately for both groups.

	**Total group (*N* = 64)**	**Negative affect group (*N* = 32)**	**Neutral affect group (*N* = 32)**
Age (years), M ± SD	24.94 ± 4.92	24.97 ± 5.19	24.91 ± 4.73
BMI (kg/m^2^), M ± SD	23.60 ± 2.89	23.12 ± 2.70	24.08 ± 3.03
Partnership (yes), *n* (%)	27 (42.2)	11 (34.4)	16 (50.0)
Smoking (yes), *n* (%)	16 (25.0)	8 (25.0)	8 (25.0)
AUDIT sum score, M ± SD	7.75 + 4.62	8.25 + 4.77	7.25 + 4.49
Pornography use (h/month) M ± SD	6.29 ± 6.61	4.99 ± 4.11	7.58 ± 8.27
s-IATsex, M ± SD	19.89 ± 5.09	19.97 ± 4.46	19.81 ± 5.73
s-IATsex > 30, *n* (%)	3 (4.7)	0 (0)	3 (9.4)
**TSMQ, M** **±** **SD**			
Solitary sexuality	3.44 ± 0.81	3.33 ± 0.74	3.56 ± 0.88
Importance of sex^*^	3.68 ± 0.76	3.49 ± 0.88	3.87 ± 0.58
Seeking sexual encounters	1.27 ± 1.05	1.03 ± 0.92	1.51 ± 1.12
Comparison with others	1.68 ± 1.09	1.51 ± 1.02	1.85 ± 1.15
Total score^*^	2.52 ± 0.66	2.34 ± 0.67	2.70 ± 0.61
**Sexual image, M** **±** **SD**			
Valence	5.67 ± 1.03	5.52 ± 1.13	5.82 ± 0.91
Arousal	3.42 ± 1.67	3.29 ± 1.63	3.55 ± 1.72
Sexual arousal	4.33 ± 1.86	4.06 ± 1.97	4.60 ± 1.74
**Neutral image, M** **±** **SD**			
Valence	5.05 ± 0.66	5.03 ± 0.69	5.08 ± 0.64
Arousal^*^	1.70 ± 1.01	1.43 ± 0.78	1.98 ± 1.13
Sexual arousal	1.05 ± 0.09	1.03 ± 0.06	1.07 ± 0.11

*BMI, Body Mass Index in kilograms per weight squared; s-IATsex, short version of the Internet Addiction Test adapted to online sexual activities; TSMQ, Trait Sexual Motivation Questionnaire.*

* *p < .05*.

### Manipulation Check

**Figure 2:**
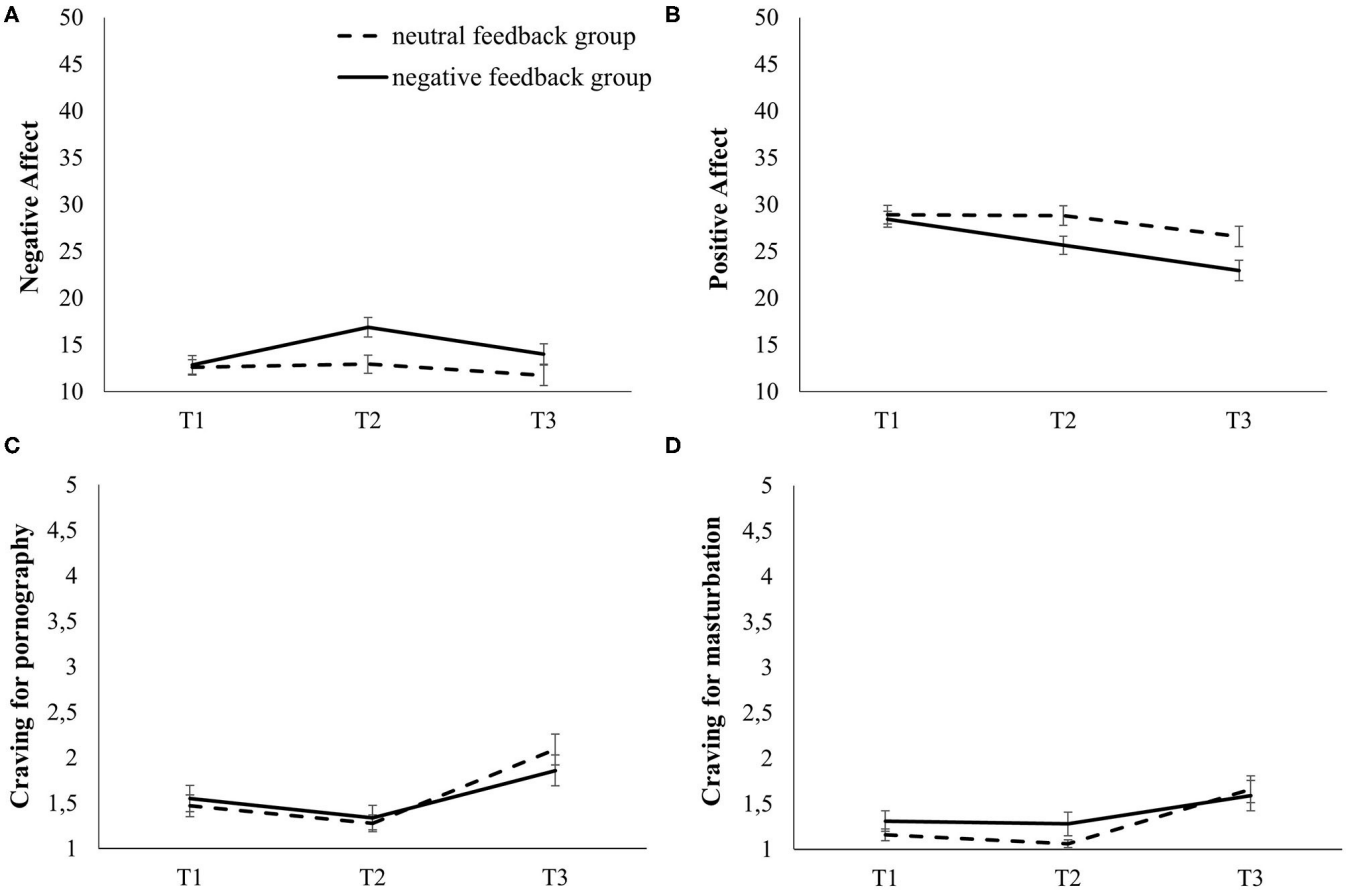
Time courses for affect and craving in both groups over the three measurement time points of the experiment. **(A)** Negative affect, **(B)** positive affect, **(C)** craving for pornography, **(D)** craving for masturbation. T1: baseline; T2: after the affect manipulation; T3: after the sexual cue reactivity paradigm.

Performance-based feedback was believed by significantly fewer people in the negative feedback group (*N* = 22; 68.8%) than in the neutral feedback group [*N* = 31; 96.9%; ${\text{X}}_{\left(1\right)}^{2}$ = 8.89, *p* = .003, φ = 0.373]. There was no difference in baseline negative affect between the groups [*t*_(62)_ = −0.44, *p* = .665], but we found an increase in negative affect [*F*_time(1.7, 108.7)_ = 15.65, *p* < .001, $\eta_{p}^{2}$= 0.202], particularly in the negative feedback group [$F_{\text{tim}{\text{e}}^{*}\text{group}\left(1.7,108.7\right)}$ = 8.94, *p* < .001, $\eta_{p}^{2}$= 0.126]. Subsequent simple contrasts confirmed an increase from baseline to post-feedback, and a return to baseline level after the sexual cue reactivity paradigm in the negative feedback group while levels in the neutral feedback group appeared stable (see Figure 2A). Regarding the manipulation of positive affect, there was no difference in baseline levels between groups [*t*_(62)_ = 0.36, *p* = .720]. We found a decrease in positive affect [*F*_time(2.0,122.8)_ = 32.75, *p* < .001, $\eta_{p}^{2}$= 0.346], particularly in the negative feedback group [$F_{\text{tim}{\text{e}}^{*}\text{group}\left(2.0,122.8\right)}$ = 6.14, *p* = .003, $\eta_{p}^{2}$= 0.090]. Subsequent simple contrasts confirmed a decrease from baseline to post-feedback, and still declining levels after the sexual cue reactivity paradigm in the negative feedback group. Levels in the neutral feedback group appeared stable from baseline to post-feedback, but there was a decline after the sexual cue reactivity paradigm (see Figure 2B).

The analyses regarding craving for pornography (of note: 3 missings in the negative feedback group) revealed small but significant changes over time [*F*_time(1.6, 96.7)_ = 28.71, *p* < .001, $\eta_{p}^{2}$ = 0.327] without differences between the feedback groups [$F_{\text{tim}{\text{e}}^{*}\text{group}\left(1.6,96.7\right)}$ = 1.92, *p* = .160, $\eta_{p}^{2}$= 0.031]. Simple contrasts confirmed a small decrease from baseline to post-feedback, but an increase after the sexual cue reactivity paradigm (Figure 2C). There were small but significant changes over time regarding the craving for masturbation [of note: 3 missings in the negative feedback group; *F*_time(1.4, 84.5)_ = 16.65, *p* < .001, $\eta_{p}^{2}$= 0.220] without differences between the feedback groups [$F_{\text{tim}{\text{e}}^{*}}\text{group}\left(1.4,\;84.5\right)$ = 1.55, *p* = .220, $\eta_{p}^{2}$= 0.026]. Subsequent simple contrasts indicated stable levels from baseline to post-feedback, but an increase after the sexual cue reactivity paradigm (Figure 2D).

### Sexual Cue Reactivity

The mixed-model ANOVA with the between-factor group (negative affect, neutral affect) and the within-factor picture category (pornographic picture, neutral picture) showed substantial stronger P300 amplitudes toward pornographic pictures compared to neutral ones [*F*_category(1.0, 62.0)_ = 362.5, *p* < .001, $\eta_{p}^{2}$= 0.854], but the groups did not differ [$F_{\text{categor}{\text{y}}^{*}\text{group}\left(1.0,62.0\right)}$ = 2.06, *p* = .157, $\eta_{p}^{2}$= 0.032; see Figure 2]. In general, the P300 levels were comparable between the groups [*F*_group(1, 62)_ = 2.58, *p* = .114, $\eta_{p}^{2}$= 0.040].

**Figure 3:**
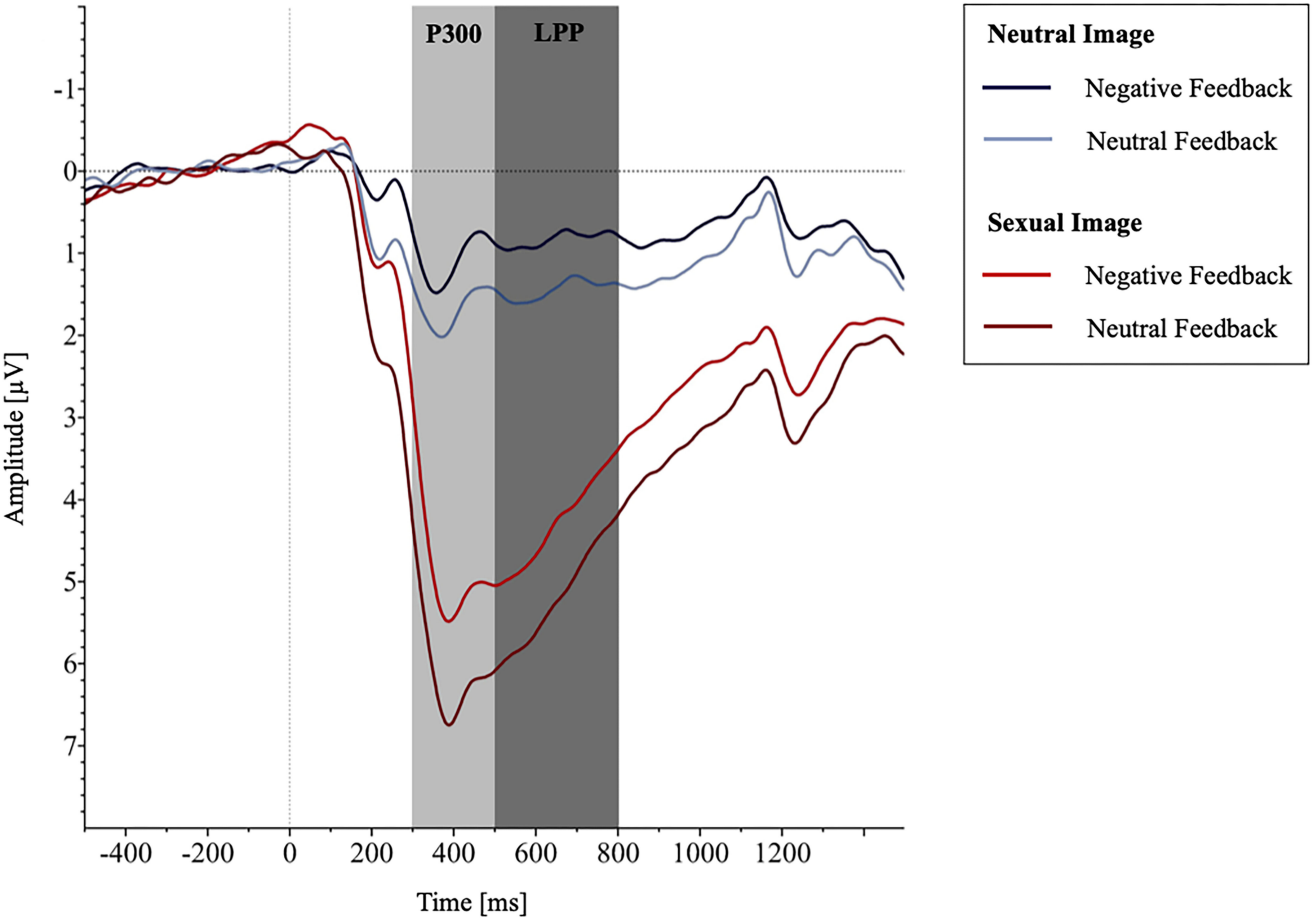
The grand average waveforms averaged across channel CP1, CP2, P3, Pz, and P4 showing the potentials produced in response to the presentation of sexual vs. neutral images depending on group allocation.

The results of the mixed-model ANOVA for LPP amplitudes displayed analogous results with substantial stronger LPP amplitude toward pornographic pictures compared to neutral ones [*F*_category(1.0, 62.0)_ = 251.20, *p* < .001, $\eta_{p}^{2}$= 0.802], no significant differences between groups [$F_{\text{categor}{\text{y}}^{*}\text{group}\left(1.0,\;62.0\right)}$ = 0.42, *p* = .522, $\eta_{p}^{2}$= 0.007; see Figure 3], and comparable LPP levels between the feedback groups [*F*_group(1, 62)_ = 2.28, *p* = .136, $\eta_{p}^{2}$= 0.036].

Compared to neutral pictures, pornographic pictures were rated as more positive, more arousing and more sexually arousing (all *F* > 15.1, *p* < .001; valence: $\eta_{p}^{2}$= 0.196; arousal: $\eta_{p}^{2}$= 0.519; sexual arousal: $\eta_{p}^{2}$= 0.767). None of the picture ratings differed between the groups (all *F* < 1.23, *p* > .273).

### Moderator Analyses

**Figure 4:**
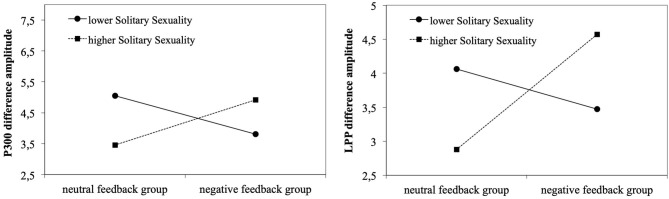
Negative feedback and solitary sexuality predicting P300 (left) and LPP amplitudes (right) toward pornographic pictures (minus neutral pictures).

Hierarchical multiple regressions indicated that neither pornography use (hours/month) nor symptoms of cybersex addiction (s-IATsex) or their interaction with group contributed significantly to the regression model (all *p* > .100, not shown). The same was true for TSMQ subscales (all *p* > .078) except the *Solitary Sexuality* subscale. Here, step-wise regression first revealed that group did not contribute significantly to the regression model, *F*_(1,62)_ = 2.06, *p* = .157, accounting for 3.2% ($R_{\text{adjusted}}^{2}$ = 0.017) of the variation in P300 difference amplitude. Introducing *TSMQ Solitary Sexuality* did not explain significant additional variation (2.3%), *F*_(1,61)_ = 1.51, *p* = .224. When the interaction was added in stage three of the regression model, the interaction was predictive of the P300 difference amplitude. Together, the predictors accounted for 15.0% ($R_{\text{adjusted}}^{2}$ = 0.108) of the variance in P300 difference amplitude, *F*_(3,60)_ = 3.54, *p* = .020 (Table 2). Likewise, LPP difference amplitudes were not predicted by the groups. The stage one regression model was not significant, *F*_(1,62)_ = 0.42, *p* = .522, accounting for 0.7% ($R_{\text{adjusted}}^{2}$ ≤ 0.001) of the variation. No significant additional variation (4.4%) was explained when adding TSMQ Solitary Sexuality, the change in *R*^2^ was not significant, *F*_(1,61)_ = 2.36, *p* = .129. In step 3, the interaction was predictive of the LPP difference amplitude and all three predictors explained 10.6% ($R_{\text{adjusted}}^{2}$ = 0.061) of the variation in LPP difference amplitudes, *F*_(3,60)_ = 2.36, *p* = .080. Figure 4 illustrates that in the negative feedback group, men with higher solitary sexual motivation levels showed higher P300/LPP difference amplitudes compared to men scoring lower on this subscale. This was contrasted by results for the neutral feedback group. Here, higher solitary sexuality was related to lower difference amplitudes.

**Table 2:** Multiple regression on late positive potential amplitudes for pornographic minus neutral pictures based on TSMQ solitary sexuality.

	***B***	***SE***	**β**	***p***	***R^**2**^***	***R*^**2**^ change**
**P300**						
*Step 1*					0.032	0.032
Group	−0.620	0.432	−0.179	0.157		
*Step 2*					0.055	0.023
Group	−0.620	0.430	−0.179	0.155		
Solitary Sexuality	0.331	0.269	0.153	0.224		
*Step 3*					0.150	0.095^*^
Group	−0.620	0.412	−0.179	0.137		
Solitary Sexuality	−0.235	0.338	−0.109	0.489		
Interaction	1.351	0.522	0.404	0.012		
**LPP**						
*Step 1*					0.007	0.007
Group	−0.029	0.457	−0.082	0.522		
*Step 2*					0.044	0.037
Group	−0.294	0.452	−0.082	0.518		
Solitary Sexuality	0.435	0.283	0.193	0.129		
*Step 3*					0.106	0.062^*^
Group	−0.294	0.441	−0.082	0.507		
Solitary Sexuality	−0.042	0.362	−0.019	0.908		
Interaction	1.138	0.559	0.384	0.046		

*N = 64; Group is coded neutral feedback = 0 and negative feedback = 1; B, unstandardized coefficient; SE, standard error of B; β, standardized coefficient; P300, positive potential 300–500 ms post stimulus; LPP, late positive potential 500–800 ms post stimulus.*

* *p < .05*.

## Discussion

In this healthy and heterosexual male sample, results confirmed the effectiveness of both negative affect manipulation and sexual cue reactivity as indicated by time-dependent changes in self-reported affect and craving ratings. While there were stronger neural responses toward pornographic pictures as compared to neutral ones, there was no effect of negative feedback. Of the moderators examined, only *Solitary Sexuality* as an aspect of trait sexual motivation had a moderating effect on the neural response, whereas symptoms of cybersex addiction or the extent of pornography use were unrelated. In the neutral feedback group, individuals with higher levels of *Solitary Sexuality* showed smaller amplitude differences of the P300 and the LPP to pornographic pictures in relation to neutral pictures, whereas the opposite was true for individuals with higher levels of *Solitary Sexuality* in the negative feedback group.

The significant increase in negative affect and decrease in positive affect following negative performance feedback provide significant evidence that the affect manipulation succeeded in a hypothesis-consistent way. In both groups, there was a decrease in craving for pornography and masturbation after performance feedback and a slight increase after the sexual cue reactivity paradigm. However, the groups did not differ in affect and craving responses. This may be traced back to a bottom effect and relatively low scores for negative affect and craving, respectively. Despite this rather low subjectively rated cue reactivity, stronger positive ERP components (for both the P300 and LPP) emerged in response to pornographic pictures compared to neutral pictures. This was consistent with our hypotheses and with prior studies (Schupp et al., 2004, 2006; Hajcak et al., 2010). Both the LPP and the P300 are discussed as indicators of motivational attention (van Lankveld and Smulders, 2008; Steele et al., 2013; Prause et al., 2015a), which in this study argues for higher attention toward pornographic pictures compared to neutral pictures. Correspondingly, pornographic pictures were rated as more pleasant, more arousing, and more sexually arousing than neutral pictures. However, we did not find significant differences between the two feedback groups (neutral, negative) for either ERPs or picture ratings. Consequently, negative affect induction had no significant effect on motivational attention or subjective evaluation of pornographic pictures in our study.

The findings that negative affect has no effect on ERPs and subjective ratings when viewing pornographic pictures complements the findings of previous studies using other methods. Carvalho et al. (2017) also found no effect of negative affect on visual attention toward pornographic pictures, assessed via eye-movement tracking, in a healthy sample of men and women. In addition, Janssen et al. (2020) found no effects of negative affect induction on physiological (penile tumescence), nor on subjective sexual arousal in hypersexual as well as non-hypersexual homosexual men. These null findings may be related to the flexibility of coping mechanisms in healthy participants, so that an affect-induced effect on the attention toward and the rating of pornographic pictures is only evident in samples with a more severe clinical expression of CSBD. From previous studies, it could be argued that negative affective states, like depression, generally reduce sexual interest and sexual cue reactivity (Bancroft et al., 2003). In some individuals, however, negative affect increases sexual behaviors, what may thus be argued to constitute a symptom of CSBD. Similar to this, missing effects of negative mood on penile tumescence in the Janssen study are discussed to be due to the sample's heterogeneity or there may be subgroups of hypersexual men with different underlying psychopathological mechanisms.

The results of the exploratory hierarchical multiple regressions revealed that neither the extent of pornography use, nor symptoms for cybersex addiction explained neural variance, i.e., neural responses toward pornographic pictures minus neutral pictures. This finding is consistent with previous study results. In their study investigating individuals with self-reported addictive pornography use, Steele et al. (2013) also found that the P300 did not correlate with addictive pornography use. In another study, participants with self-reported addictive pornography use showed lower LPP amplitudes than control participants (Prause et al., 2015b). However, the latter study involved participants who reported relatively low levels of addictive pornography use who either already had less pronounced levels from the onset of their addiction or had already reduced their use. Another study from Stark et al. (2019) showed no significant correlations between the level of pornography use and neural responses to sexual stimuli in young health men and women. The authors discuss that the processing of sexual stimuli in healthy individuals may be subject to a strong evolutionary biological influence. Given previous assumptions on addictive pornography use, we would have expected a correlation between hypersensitivity to pornographic stimuli and measures of cybersex addiction (Voon et al., 2014; Brand et al., 2019). But sexual traits such as habitual pornography use or addictive symptoms may only slightly influence the processing of sexual stimuli in healthy individuals and clinical samples would be needed to investigate these abnormalities. Of note, the scores for cybersex addiction in the current study are comparable to the scores in the validation study (Laier et al., 2013). Three participants (all from the neutral feedback group) reported values that, according to Pawlikowski et al. (2013), indicate problematic pornography use. This result creates variance between groups that is not attributable to the affect manipulation and thus reduces its effect. As a note, excluding these individuals did not change mixed-model ANOVA findings or regression analyses of the moderators pornography use and cybersex addiction.

For trait sexual motivation, the current study showed a significant interaction between the *Solitary Sexuality* subscale of the TSMQ and group membership (neutral feedback group vs. negative feedback group) with regard to the amplitude difference between pornographic and neutral pictures. In the neutral feedback group, individuals with higher expression of *Solitary Sexuality* responded with smaller amplitude differences (pornographic picture minus neutral picture) of the P300 and the LPP component, whereas in the negative feedback group, individuals with higher expression of Solitary Sexuality responded to pornographic pictures with higher amplitude differences of the P300 and the LPP. A higher amplitude difference could be an indicator of sexual hyperresponsiveness, whereas a lower amplitude difference could indicate sexual hyporesponsiveness. The *Solitary Sexuality* subscale consists of items assessing the relationship-independent interest in pornography or sexual fantasies and interest in sexual activities such as masturbation (Stark et al., 2015). The interaction result suggests that individuals with higher levels of *Solitary Sexuality* may present stronger motivational attention to pornographic stimuli during negative affect, which could be indicative of higher sexual reward sensitivity. Whether such a link between higher relationship-independent interest in sexual activities and hyperresponsivity to pornographic stimuli under negative affect underlies addictive pornography use, has now to be shown in clinical samples. Interestingly, the graphic illustration of this interaction also indicated that men scoring lower on *Solitary Sexuality* responded with higher amplitude differences under neutral feedback. For these men, pornographic images seem to represent a stronger motivationally relevant stimulus than for men with a higher relationship-independent interest in sexual activities. Overall, the effect of negative affect on cue reactivity was found to dependent on the level of *Solitary Sexuality*. At low levels of this trait, negative affect has a dampening effect on cue reactivity, while at higher levels it has an enhancing effect. The result of the relationship between the neural correlates and sexual motivation is consistent with findings of previous fMRI studies. Stark et al. (2019) found a positive association between sexual motivation and neural responses to the contrast between sexual and neutral stimuli in the occipital/parietal region. Consistent with this, the study of Strahler et al. (2018) reported positive associations between sexual motivation and caudate nucleus activity. What needs to be added here is that the exclusion of the three men self-reporting problematic pornography use led to an in this case significant main effect of TSMQ *Seeking Sexual Encounters* on LPP difference amplitudes (*B* = −0.671, β = −0.354, *p* = .045). Individuals with higher expression of *Seeking Sexual Encounters* responded with smaller amplitude differences possibly indicating sexual hyporesponsiveness. We do not want to interpret this post analysis too prominently but a process of habituation to greater sexual experiences may explain this (Bancroft et al., 2009).

### Limitations and Suggestions for Future Studies

We have to mention some important limitations of the current study. Since participants completed the trait questionnaires after the cue reactivity paradigm, it cannot be ruled out that the affect induction had an impact on the completion of the questionnaires. This should be avoided in future studies by changing the study procedure accordingly. In addition, it is possible that viewing pornographic material evokes feelings of shame (or other negative feelings) in recipients which then again may impact how pornographic material is attended to (Maskeliunas and Raudonis, 2016). For ethical reasons, it was mentioned at the beginning of the recruitment process that this study is about the processing of sexual images. This may have introduced some selection bias as men who feel, e.g., ashamed when watching pornographic material would not participate. Whether the current sample felt ashamed throughout testing has not been evaluated, though the pornographic stimuli were rated with an average valence of 5.67 indicating neutral to positive feelings. While shame should not have played a role in the testing of our hypotheses, this may limit validity of findings for the general population. The generalizability of the results is also limited by the fact that this study focused on an all-male, healthy, heterosexual sample given the known gender differences in habitual pornography consumption, experienced problems with pornography consumption, and gender-specific interactions between negative affect and sexual reactivity (Cooper et al., 1999; Hald, 2006; Lykins et al., 2006; Ross et al., 2012; Carvalho et al., 2017). In addition, pornographic pictures depicted heterosexual content due to known effect of sexual orientation on brain responses toward pornographic pictures (Paul et al., 2008). Moreover, the laboratory setting may not only limit the transferability of the results to everyday life but could also bias self-report data (e.g., shame, social desirability). The choice of the affect induction can be regarded as a strength. Comparable to everyday situations, the performance test involves ego-involvement of the participants (Nummenmaa and Niemi, 2004) and compared to other affect induction methods (e.g., music, videos) they did not know that their affect was manipulated. At the end of the survey, significantly more participants from the negative feedback group compared to the neutral feedback group reported that they did not believe the cover story. The hypothesis-compliant decrease in positive affect and increase in negative affect nevertheless suggest that the cover story even if doubted still affected the participants. A possible explanation for these contradictory data could be that the questioning of the cover story occurred later. Manipulating task difficulty instead of manipulating feedback may improve the credibility of such cover stories in future studies (Nummenmaa and Niemi, 2004).

### Conclusions

The results support a role for solitary sexual motivation in motivated attention toward pornographic pictures among men receiving negative performance feedback. From this findings, some aspects of trait sexual motivation might be understood as factors predisposing to increased motivated attention toward pornographic cue stimuli under negative mood. Findings partly support theories which suggest pornography use to be increasingly used for emotion regulation, which then becomes a reinforcing or perpetuating mechanism for repeated and possibly increased pornography use. Other characteristics of sexual behaviors and traits provided no exploratory value and there was no general effect of negative affect in the processing of pornographic pictures. Since aspects such as the extent of pornography consumption or symptoms of cybersex addiction only become of relevance at a clinical stage, patient studies are necessary to explore neurophysiological mechanisms of CSBD. Overall, findings suggest reinforcing effects of pornography use and contribute to our understanding of neuropsychological mechanisms of sexual cue reactivity and addiction development.

## Data Availability Statement

The dataset presented in this study can be found in an online repository. The name of the repository and accession link (URL), and doi can be found below: JLUpub http://dx.doi.org/10.22029/jlupub-12.2.

## Ethics Statement

The studies involving human participants were reviewed and approved by the local ethics committee of the Department of Psychology and Sports Science, University of Giessen, Giessen, Germany (reference number: 2019–0005). The patients/participants provided their written informed consent to participate in this study.

## Author Contributions

JS, SK, and RS conceived the study. SK collected the data. JS, SK, and CM analyzed the data and drafted the manuscript. JS, SK, AB, CM, and RS interpreted the data and revised the article for important intellectual content. All authors contributed to the article and approved the submitted version.

## Conflict of Interest

The authors declare that the research was conducted in the absence of any commercial or financial relationships that could be construed as a potential conflict of interest.
